# The association between obesity and problematic smartphone use among school-age children and adolescents: a cross-sectional study in Shanghai

**DOI:** 10.1186/s12889-021-12124-6

**Published:** 2021-11-11

**Authors:** Zhicong Ma, Jiangqi Wang, Jiang Li, Yingnan Jia

**Affiliations:** 1 School of Public Health, Key Lab of Public Health Safety of the Ministry of Education, Fudan University, Shanghai, 200032 China; 2 Health Communication Institute, Fudan University, Shanghai, 200032 China

**Keywords:** Obesity, Problematic smartphone use, Children, Adolescents

## Abstract

**Background:**

The study aimed to investigate the association between content-based problematic smartphone use and obesity in school-age children and adolescents, including variations in the association by educational stage and sex.

**Methods:**

Two-stage non-probability sampling was used to recruit 8419 participants from nineteen primary schools, five middle schools, and thirteen high schools in Shanghai in December 2017. Obesity was identified by body mass index (BMI), which was obtained from the school physical examination record, while problematic smartphone use was measured by the Revised Problematic Smartphone Use Classification Scale as the independent variable.

**Results:**

The rates of obesity varied with educational stages, while problematic smartphone use increased with educational stages. Male students reported higher obesity rates (37.1%vs19.4%, *P* < 0.001) and greater problematic smartphone use scores (25.65 ± 10.37 vs 22.88 ± 8.94, *P* < 0.001) than female students. Problematic smartphone use for entertainment (smartphone users addicted to entertainment games, music, videos, novels and other applications) was positively associated to obesity status for primary school [odds ratio (OR), 1.030; 95% confidence interval (95% CI), 1.005–1.057] and high school students (OR, 1.031; 95% CI, 1.004–1.059). For female students, problematic smartphone use for entertainment was positively associated with obesity status (OR, 1.046; 95% CI, 1.018–1.075).

**Conclusions:**

Problematic smartphone use may be associated with obesity in children and adolescents. The association differed based on the educational stage and sex, and the difference possessed dimensional specificity.

**Supplementary Information:**

The online version contains supplementary material available at 10.1186/s12889-021-12124-6.

## Background

Overweight and obesity in children and adolescents have been named a worldwide public health problem. Among children and adolescents aged 5–19 years around the world, more than 340 million were reported to be overweight in 2016, and the prevalence has continued to rise dramatically [1]. Similarly, the prevalence of overweight/obesity among children aged 0–18 in China increased from 5.0%/1.7% in 1991–1995 to 11.7%/6.8% in 2011–2015, respectively [2, 3]. Research shows that most of these obese adolescents show varying degrees of decreased self-esteem, accompanied by related mental health problems, such as anxiety, loneliness, and high-risk behaviors [3, 4]. This situation even has potential adverse effects on their health in adulthood [5]. For example, overweight in childhood is associated with increased adult all-cause and cardiovascular mortality [6]. The correlates of obesity in children and adolescents are numerous and complex but have already been demonstrated to include a lack of physical activity, sedentary behavior, increased screen time, and mental health [7–10]. And the risk of overweight/obesity among adolescents was significantly positively associated with sedentary behavior and negatively associated with mental health.

China has developed into one of the world’s largest smartphone markets, as modern electronic devices increasingly intrude into daily life [11]. With the popularization of smartphones, the time people spend on the Internet has dramatically increased, with young Internet users, in particular, being especially prevalent [12–14]; 37.9% of the Chinese netizen population is middle school students, while 25.4% encompasses other types of school students [14]. A national survey on Internet dependence (a sense of dependence on the Internet due to bad or excessive use) among adolescents in China revealed that the Internet dependence rate is 7%, albeit ranging from 6 to 12% in different provinces [15]. Students of school age have become the largest group of Internet users in China [14]. Because adolescents are the most highly affected by and at risk for both substance and behavioural addition due to their lack of self-control, they are more susceptible to the adverse outcomes of smartphone use than others [16].

Despite the benefits offered by smartphones [e.g., the optimization of communication, development of health promotion interventions, and applications] [17–19], self-reported smartphone dependence, together with addiction-like symptoms, represents a significant global health concern that should not be neglected [20–22]. In many countries, it is estimated that the prevalence rate of smartphone dependence is 10–30% among adolescents, and this rate is still experiencing rapid growth [23]. So it is necessary to understand the potential problem caused by smartphone dependence [24].

Several studies have indicated a possible association between smartphone dependence and childhood obesity, pointing out that excessive usage of smartphones serves as a potential risk factor of obesity [25, 26]. A study cross-sectional designed to investigate the association between excessive smartphone use on the physical activity of 110 Chinese international students aged 19–25 years old shows that smartphone dependence may affect physical health and thus result in an increase in one’s fat mass by reducing physical activity [27]. Another domestic research study suggests that excessive use of smartphones by Chinese children and adolescents is also contributing to obesity due to sedentary screen time and snack intake [28, 29]. The Chinese 2005 NYRBS (National Youth Risk Behavior Surveillance) reported that about 30% of Chinese boys and 15% of Chinese girls spent > 2 h per day playing electronic games including mobile games and computer games [30]. This situation will become worse due to the popularity of smart phones. Research shows that problematic smartphone use is significantly associated with loneliness [31], low self-esteem, and depression [16]. Poor mental health is also proven to be a risk factor for childhood obesity [32]. And smartphone dependence causes adverse effects on one’s lifestyle such as dietary habits and daily routines, resulting in overweight or obesity [33]. Additionally, academic stress also contributes to addictive behaviors, such as smartphone addiction [34]. Based on the influencing factors of childhood obesity provided by the Mayo Clinic [35], health behaviors including physical activity, sedentary behavior, and screen time—together with mental health and academic stress, which have also been linked to problematic smartphone use—were collected as covariates in the present study [16]. Due to the distinct differences in physical activities, mental health, smartphone usage patterns, and academic stress among different sex and educational stages, the correlation of problematic smartphone use and BMI may also differ based on sex and educational stage [16]. Although there have been studies focusing on the correlation between problematic smartphone use and BMI, the association between content-based problematic smartphone use and BMI among children with school-age and adolescents has rarely been studied. Moreover, in the context of the COVID-19 pandemic, the frequency and duration of smartphone use may increase. Christoffer Clemmensen et al. voiced concern about the possibility that the social strategies implemented to oppose COVID-19 might have long- term, negative effects on the obesity epidemic [36]. In China, due to the wide application of online education during the COVID-19 pandemic, the time and frequency of smartphone use may increase more among children and adolescents [37].

Our study, therefore, sought to investigate the direct association between content-based problematic smartphone use and overweight/obesity (as measured by BMI) in Chinese children with school-age and adolescents using a cross-sectional design.

## Methods

### Sample and procedure

This research was conducted in nineteen primary schools, five middle schools, and thirteen high schools in Shanghai between December 14, 2017, and December 27, 2017. Two-stage non-probability sampling was performed to enroll participants. At the first stage, thirty-seven schools in Shanghai with available participants were selected, including nineteen primary schools, five middle schools, and thirteen high schools. For each of these schools, students were recruited at the second stage based on the following rules: For primary schools, students were selected from the fourth and fifth grades, while middle school and high school students were selected from the seventh and tenth grades. The distribution of the participants across different educational stages and districts is presented in Table S1. The questionnaire survey was organized by school health care teachers and head teachers. Participants and their parents were asked to complete written informed consent forms. If the participants themselves or their parents disagree, the participants will not be included. A total of 8360 (99.3%) of 8419 eligible participants provided complete data. This study received approval from the ethics committee of the School of Public Health of Fudan University, China (IRB2018120723 and FWA0002399).

### Measures

#### Measures of BMI

Bodyweight and height were obtained from the school physical examination record. During school physical examination, all technicians had been trained for the recording of anthropometric measurements, and students were asked to wear light clothes, be barefoot, and stand straight. Bodyweight was measured to the nearest 0.1 kg, and height was measured to the nearest 0.1 cm. The bodyweights were measured uniformly using German imported Seca-877 electronic weight scales. BMI was calculated as the ratio of weight in kilograms to the square of height in meters. Based on the BMI-based age and sex-specific criteria provided by the Working Group on Obesity in China (WGOC), [38] participants were classified into four groups, as follows: underweight, normal, overweight, and obese.

#### Development of revised problematic smartphone use classification scale (RPSUCS)

The Revised Problematic Smartphone Use Classification Scale (RPSUCS), which was developed on the characteristics of Chinese urban and suburban school-age children and adolescents, using the Problematic Smartphone Use Classification Scale (PSUCS) by Hu as a reference, [39] was used to measure problematic smartphone use in all participants. Permission to use the scale was obtained from its author. Internal consistency reliability was measured using the Cronbach α coefficient. The Cronbach’s α coefficient of the total scale of RPSUCS, problematic smartphone use on social network, entertainment, and information collection were 0.876, 0.863, 0.852, and 0.750, respectively. The test-retest reliability of RPSUCS was assessed in 540 participants two months later by Interclass Correlation Coefficient (ICC) of the total score and the score of three dimensions at the two tests. The ICCs of the total scale, problematic smartphone use on social network, entertainment, and information collection were 0.763, 0.850, 0.645, and 0.609, respectively. Exploratory factor analysis extracted a three-factor structure accounting for 65.074% of the total variance. The following Goodness of Fit indices indicated a good fit of the proposed three-factor model of the RPSUCS to the data: Chi-square (χ^2^) = 1490.915, *p* < 0.001, NFI = 0.969, RFI = 0.960, IFI = 0.970, TLI = 0.962, CFI = 0.970, and RMSEA = 0.053. See supplementary materials S2 for more details. Investigators had been trained to explain the items for middle and primary school students’ interpretation if needed.

#### Measures of problematic smartphone use

Problematic smartphone use was stratified into three dimensions in the RPSUCS, as follows: “Social network,” “entertainment,” and “information collection.” A total of thirteen items of the RPSUCS are presented in Table S3. Participants rated all items on a five-point continuum scale, as follows: 1 = strongly disagree, 2 = disagree, 3 = neither agree nor disagree, 4 = agree, and 5 = strongly agree. Items of the same dimension were averaged to obtain the subscale score. A high subscale score indicated high problematic smartphone use in that dimension. Six items were used to measure social network with a total score of 30 points, of which an example was, “Parents and friends complained about the time spent surfing the Internet, but I still did not reduce the time spent online.” Entertainment was assessed by four items, with a total possible score of 20 points, of which an example was, “Using entertainment apps (e.g., Internet novels, games, music, video and other applications) is my favorite way to relax and reduce stress.” The three-item information collection subscale had a total score of 15 points, with an example being “If I do not read news, I will feel restless.”

#### Measures of covariates

Data on demographic characteristics (e.g., sex, age, and educational stage), health behaviors (e.g., indoor physical activity time, outdoor physical activity time, sedentary time, and screen time), mental health, and academic stress were collected by self-report questionnaire from all participants. Investigators had been trained to explain the items for middle and primary school students, and students were able to ask for clarification during the investigation.

For health behaviors, indoor and outdoor physical activity times were measured by asking how long the participant performed indoor and outdoor (including walking and cycling) physical activity per day in the recent school term, with the following five possible options provided as answers: < 10, 10–30, 30–60, 60–90, and > 90 min per day. Sedentary time was measured by asking, “What was your average sedentary time per day,” with five possible answers being < 2, 2–4, 4–6, 6–8, and > 8 h per day. Participants were also required to report the hours they spent on a smartphone, computer, tablet, and other electronic devices each day. Non-smartphone screen time was the sum of the screen times of said other electronic devices.

We used the Chinese version of the World Health Organization’s Five-item Well-being Index (WHO-5) to evaluate participants’ mental health conditions [40]. Participants were asked to rate five statements according to their applicability in the last two weeks using a score range of 0 = at no time to 5 = all of the time. A sample statement is, “I have felt active and vigorous.” The total score for the WHO-5 ranged from 0 to 25 points. Participants with total scores of less than 13 points were classified as being in “poor mental health,” while those with total scores of greater than or equal to 13 points had “good mental health.” Academic stress was measured on a five-point scale with one question: “Would you say that your study pressure is very much, much, moderate, a little, or none?”

### Statistical analyses

Due to the low proportion of indoor and outdoor physical activity times of less than 10 or greater than 90 min per day, indoor and outdoor physical activity time categories were reclassified as either < 30, 30–60, or > 60 min per day. A descriptive analysis was performed on continuous variables (e.g., academic stress and problematic smartphone use) and categorical variables (e.g., sex and BMI). The association between BMI and continuous variables was quantified by analysis of variance, and the comparison between BMI and categorical variables was obtained via the chi-squared test grouped by educational stage and sex, respectively. The referent level of sex, indoor physical activity time, outdoor physical activity time, and mental health were set to be “male,” “< 30,” “< 30,” and “poor.” Since there were too few observations of underweight participants (6.6%) compared with normal participants (65.2%), BMI was classified into the following two groups: underweight/normal and overweight/obese. Since this study needs to include all variables (including research variables and other possible influencing factors) into the model at one time and make corrections, the multivariate logistic regression model using the “Enter” method was adopted to investigate the relationship between problematic smartphone use and BMI, adjusted for sex, age, educational stage, mental health, physical activity, academic stress, and non-smartphone screen time. Participants with no smartphone screen time (indicating no access to their smartphones) were screened out in the multivariate logistic regression models. All statistical tests were two-sided, and a *p*-value of less than 0.05 was considered as the cut-off value for statistical significance. Analyses were performed using the Statistical Package for Social Sciences version 25 (IBM Corp., Armonk, NY, USA).

## Results

Table 1 shows that of the 7506 participants with complete data, 3732 (49.7%) were male, and 3774 (50.3%) were female. The average age of participants was 12.43 years old. The result showed that 37.1% of male students were found to be overweight or obese, while 19.4% of females were overweight or obese. And the corresponding problematic smartphone use scores (out of 65 points) were 25.65 ± 10.37 and 22.88 ± 8.94 points. In comparison with students in middle school and high school, the proportion of obesity among students in primary school was highest. Except for the middle school group, the proportion of overweightness/obesity was higher in male students than in female students in the primary and high schools. At different educational stages, the total score of PSU of male students was higher than that of female students (*p* < 0.001). Significant differences (p < 0.001) were shown in the distribution of all variables among different educational stages. PSU, academic stress, and poor mental health went higher with the improvement of educational stage.

**Table 1:** Demographic characteristics and potential overweight/obesity influencing factors of participants of different genders at different educational stages

		Primary school			χ^2^ (p) / F (p)	Middle school			χ^2^ (p) / F (p)	High school			χ^2^ (p) / F (p)	χ^2^ (p) / F (p)
		Overall n (%)	Male n (%)	Female n (%)		Overall n (%)	Male n (%)	Female n (%)		Overall n (%)	Male n (%)	Female n (%)		
Age		10.17 ± 0.61	10.17 ± 0.62	10.16 ± 0.60	0.93	13.18 ± 0.92	13.22 ± 0.96	13.14 ± 0.86	1.29	16.27 ± 0.66	16.27 ± 0.70	16.28 ± 0.63	−0.22	68,865.33 ^***^
Height (cm)		143.85 ± 7.87	144.13 ± 7.59	143.57 ± 8.14	2.33^*^	161.72 ± 8.14	163.95 ± 8.44	159.04 ± 6.89	9.08^***^	168.22 ± 8.45	174.47 ± 6.32	162.78 ± 5.93	46.41^***^	7222.47 ^***^
Weight (kg)		38.81 ± 9.73	40.42 ± 10.33	37.16 ± 8.78	11.13^***^	53.02 ± 12.01	54.53 ± 12.97	51.21 ± 10.47	4.01^***^	62.27 ± 14.13	69.22 ± 14.62	56.24 ± 10.45	25.09^***^	2820.37 ^***^
Body mass index (kg/m^2^)	Underweight	229 (5.3)	92 (4.2)	137 (6.4)	221.64^***^	65 (7.8)	41 (9.0)	24 (6.3)	7.69	198 (8.4)	71 (6.4)	127 (10.0)	94.39^***^	54.10 ^***^
	Normal	2796 (65.0)	1216 (55.9)	1580 (74.3)		563 (67.4)	289 (63.4)	274 (72.3)		1538 (64.9)	638 (57.9)	900 (70.9)		
	Overweight	667 (15.5)	466 (21.4)	201 (9.5)		128 (15.3)	79 (17.3)	49 (12.9)		400 (16.9)	229 (20.8)	171 (13.5)		
	Obese	608 (14.1)	400 (18.4)	208 (9.8)		79 (9.5)	47 (10.3)	32 (8.4)		235 (9.9)	164 (14.9)	71 (5.6)		
Indoor PA time (m/day)^†^	< 30	2034 (47.9)	1013 (47.2)	1021 (48.6)	30.54^***^	435 (52.1)	237 (52.4)	198 (53.2)	0.34	1642 (69.3)	755 (68.9)	887 (70.3)	4.97^*^	297.85 ^***^
	30–60	1383 (32.6)	645 (30.1)	738 (35.1)		256 (30.7)	139 (30.8)	117 (31.5)		480 (20.2)	215 (19.6)	265 (21.0)		
	> 60	829 (19.5)	486 (22.7)	343 (16.3)		133 (15.9)	76 (16.8)	57 (15.3)		235 (9.9)	125 (11.4)	110 (8.7)		
Outdoor PA time (m/day)	< 30	910 (21.3)	453 (21.0)	457 (21.6)	20.88^***^	125 (15.0)	76 (16.7)	49 (12.9)	4.59	617 (26.0)	259 (23.5)	358 (28.3)	9.24^*^	184.77^***^
	30–60	1637 (38.2)	764 (35.3)	873 (41.2)		312 (37.4)	177 (38.8)	135 (35.6)		1125 (47.4)	526 (47.8)	599 (47.3)		
	> 60	1734 (40.5)	945 (43.7)	789 (37.2)		398 (47.7)	203 (44.5)	195 (51.5)		625 (26.4)	316 (28.7)	309 (24.4)		
Sedentary time (h/day)	< 2	925 (21.7)	498 (23.1)	427 (20.2)	10.25^*^	91 (10.9)	60 (13.2)	31 (8.2)	14.63^**^	86 (3.6)	59 (5.4)	27 (2.1)	33.83^***^	1507.97 ^***^
	2–4	1190 (27.7)	569 (26.4)	621 (29.4)		223 (26.7)	132 (29.0)	91 (24.0)		236 (10.0)	128 (11.6)	108 (8.5)		
	4–6	1013 (23.6)	501 (23.2)	512 (24.2)		188 (22.5)	101 (22.2)	87 (23.0)		391 (16.5)	200 (18.2)	191 (15.1)		
	6–8	705 (16.4)	352 (16.3)	353 (16.7)		159 (19.0)	86 (18.9)	73 (19.3)		573 (24.2)	240 (21.8)	333 (26.3)		
	> 8	435 (10.1)	235 (10.9)	200 (9.5)		173 (20.7)	76 (16.7)	97 (25.6)		1080 (45.6)	474 (43.1)	606 (47.9)		
Academic stress^a^		2.50 ± 1.04	2.56 ± 1.08	2.43 ± 0.99	4.04^***^	2.90 ± 0.86	2.97 ± 0.88	2.80 ± 0.81	2.90^**^	3.27 ± 0.91	3.32 ± 1.02	3.22 ± 0.80	2.72^**^	495.89 ^***^
Mental health^b^	Poor	550 (13.0)	298 (13.9)	252 (12.0)	3.66	182 (21.8)	110 (24.3)	72 (19.1)	3.29	848 (35.8)	372 (34.0)	476 (37.7)	3.56	480.73 ^***^
	Good	3694 (87.0)	1840 (86.1)	1854 (88.0)		647 (77.5)	342 (75.7)	305 (80.9)		1507 (63.6)	722 (66.0)	785 (62.3)		
Smartphone screen time (h/day)		0.61 ± 0.88	0.62 ± 0.93	0.60 ± 0.82	0.51	1.73 ± 1.72	1.78 ± 1.82	1.66 ± 1.59	1.02	1.98 ± 1.59	1.85 ± 1.56	2.09 ± 1.60	−3.68^***^	855.60 ^***^
Non-smartphonescreen time (h/day)		1.12 ± 1.74	1.26 ± 1.92	0.97 ± 1.51	5.56^***^	1.35 ± 2.17	1.58 ± 2.32	1.07 ± 1.94	3.30^**^	0.79 ± 1.49	1.02 ± 1.91	0.58 ± 0.95	6.96^***^	43.23 ^***^
PSU for social network (out of 30 points) ^‡,c^		8.01 ± 3.63	8.33 ± 4.03	7.68 ± 3.14	5.86^***^	10.65 ± 4.56	11.03 ± 4.92	10.18 ± 4.05	2.69^**^	12.97 ± 5.14	12.91 ± 5.45	13.01 ± 4.86	−0.49	908.61 ^***^
PSU for entertainment (out of 20 points)		7.56 ± 4.24	8.36 ± 4.61	6.74 ± 3.66	12.68^***^	9.85 ± 4.48	11.26 ± 4.43	8.15 ± 3.91	10.66^***^	10.77 ± 4.40	12.20 ± 4.38	9.54 ± 4.03	15.38^***^	444.86^***^
PSU for information collection (out of 15 points)		5.42 ± 3.06	5.67 ± 3.26	5.16 ± 2.81	5.42^***^	5.79 ± 2.80	6.02 ± 2.91	5.53 ± 2.64	2.49^*^	5.79 ± 2.73	6.00 ± 2.92	5.61 ± 2.54	3.40^**^	15.45^***^
Total score of PSU (out of 65 points)		20.95 ± 8.66	22.32 ± 9.43	19.54 ± 7.55	10.63^***^	26.24 ± 9.21	28.24 ± 9.47	23.83 ± 8.30	7.09^***^	29.52 ± 9.35	31.09 ± 9.87	28.16 ± 8.64	7.69^***^	703.51^***^

*P < 0.05; **P < 0.01; ***P < 0.001

† PA: Physical activity. ‡ PSU: Problematic smartphone use

a Measured on a five-point scale with one question: “Would you say that your study pressure is very much, much, moderate, a little, or none?”

b Measured on the Chinese version of the World Health Organization’s Five-item Well-being Index (WHO-5)

c Dimensions of problematic smartphone use (social network, entertainment, and information collection) were measured on the Revised Problematic Smartphone Use Classification Scale

Table 2 (categorical variables) and Table 3 (continuous variables) show the comparison between body mass index and potential overweight/obesity influencing factors of participants, respectively. In Table 2, among different educational stages, the difference was statistically significant, with the highest percentage of obesity in primary school, reaching 29.7%. The proportion of obesity in different sedentary time groups was not the same, which the 6-8 h/day group was the lowest (23.9%). In Table 3, compared with the underweight/normal participants, overweight/obese participants were younger (12.68 years old VS 12.99 years old); non-smartphone screen time was longer (1.27 h/day VS 1.07 h/day); entertainment PSU scores were higher (9.96 VS 9.20), and the total scores of PSU were higher (26.48 VS 25.48). To be specific, multivariate logistic regression models were employed to examine the direct association between problematic smartphone use and obesity status among different subgroups in Table 4 and Table 5. In Table 4, among students at different educational stages, the influencing factors of obesity were not completely the same. Among primary and high school students, female students had lower odds of overweight/obesity (OR = 0.383 and 0.483) as compared with male students. PSU for entertainment was positively correlated with obesity in both primary school (OR = 1.030) and high school (OR = 1.031). In addition, among students from primary school, age was negatively associated with obesity (OR = 0.810). But the above associations were not found among middle school students. In Table 5, the factors affecting obesity of different sexes were not completely the same. For male students, only the educational stage was associated with obesity. Students from middle school had lower odds of overweight/obesity (OR = 0.605) in comparison with students from primary school. For female students, female students from middle school (OR = 2.627) and high school (OR = 5.482) had higher odds of overweight/obesity. Age was negatively associated with obesity (OR = 0.751). The PSU for entertainment positively correlated with obesity status in female students (OR = 1.046).

**Table 2:** Comparison between body mass index and potential overweight/obesity influencing factors of participants (categorical variables)

Variables		Underweight/normal n (%)	Overweight/obese n (%)	χ^2^ (p)
Sex	Male	2347 (62.9)	1385 (37.1)	290.83^***^
	Female	3042 (80.6)	732 (19.4)	
Educational stage	Primary school	3025 (70.3)	1275 (29.7)	11.62^**^
	Middle school	628 (75.2)	207 (24.8)	
	High school	1736 (73.2)	635 (26.8)	
Indoor PA time (m/day)^†^	< 30	2948 (71.7)	1163 (28.3)	0.14
	30–60	1527 (72.1)	592 (27.9)	
	> 60	856 (71.5)	341 (28.5)	
Outdoor PA time (m/day)	< 30	1185 (71.7)	467 (28.3)	0.05
	30–60	2211 (71.9)	863 (28.1)	
	> 60	1976 (71.7)	781 (28.3)	
Sedentary time (h/day)	< 2	768 (69.7)	334 (30.3)	18.06^**^
	2–4	1188 (72.0)	461 (28.0)	
	4–6	1120 (70.4)	472 (29.6)	
	6–8	1093 (76.1)	344 (23.9)	
	> 8	1193 (70.7)	495 (29.3)	
Mental health^a^	Poor	1126 (71.3)	454 (28.7)	0.35
	Good	4212 (72.0)	1636 (28.0)	

*P < 0.05; **P < 0.01; ***P < 0.001

† *PA* Physical activity

a Measured on the Chinese version of the World Health Organization’s Five-item Well-being Index (WHO-5)

**Table 3:** Comparison between body mass index and potential overweight/obesity influencing factors of participants (continuous variables)^a^

Variables	Underweight/normal Mean (Standard deviation)	Overweight/obese Mean (Standard deviation)	F (p)	η^2^
Age	12.99 ± 2.91	12.68 ± 2.92	13.33 ^***^	0.002
Academic stress^b^	2.87 ± 0.98	2.88 ± 1.06	0.10	< 0.001
Smartphone screen time (h/day)	1.50 ± 1.41	1.55 ± 1.48	1.02	< 0.001
Non-smartphone screen time (h/day)	1.07 ± 1.66	1.27 ± 2.01	12.40^***^	0.003
PSU for social network (out of 30 points)^‡,c^	10.57 ± 4.94	10.62 ± 5.07	0.12	< 0.001
PSU for entertainment (out of 20 points)	9.20 ± 4.47	9.96 ± 4.71	30.53^***^	0.006
PSU for information collection (out of 15 points)	5.75 ± 2.92	5.92 ± 3.09	3.38	0.001
Total score of PSU (out of 65 points)	25.48 ± 9.62	26.48 ± 10.03	11.71 ^**^	0.002

*P < 0.05; **P < 0.01; ***P < 0.001

‡ *PSU* Problematic smartphone use

a Participants with non-zero smartphone screen time were recruited

b Measured on a five-point scale with one question: “Would you say that your study pressure is very much, much, moderate, a little, or none?”

c Dimensions of problematic smartphone use (social network, entertainment, and information collection) were measured on the Revised Problematic Smartphone Use Classification Scale

**Table 4:** Odds ratios (OR) and 95% confidence intervals (95% CI) of predictors of overweight/obesity in participants by educational stage^a^

Variables		Model A: *n* = 2567 Primary school OR (95% CI)	Model B: *n* = 663 Middle school OR (95% CI)	Model C: *n* = 2067 High school OR (95% CI)
Sex	Male	Reference	Reference	Reference
	Female	0.383 (0.320–0.458)^***^	0.753 (0.514–1.102)	0.483 (0.390–0.598) ^***^
Age		0.810 (0.702–0.935)^**^	0.874 (0.713–1.070)	0.933 (0.804–1.083)
Indoor PA time (m/day)^†^	< 30	Reference	Reference	Reference
	30–60	0.956 (0.774–1.180)	0.894 (0.587–1.361)	1.230 (0.959–1.577)
	> 60	0.917 (0.710–1.184)	1.296 (0.785–2.138)	0.977 (0.685–1.392)
Outdoor PA time (m/day)	< 30	Reference	Reference	Reference
	30–60	0.884 (0.700–1.115)	0.806 (0.473–1.375)	1.194 (0.922–1.545)
	> 60	0.753 (0.584–0.969)	0.809 (0.473–1.384)	1.399 (1.041–1.880)
Academic stress^b^		1.002 (0.918–1.094)	1.092 (0.876–1.362)	1.029 (0.916–1.156)
Mental health^c^	Poor	Reference	Reference	Reference
	Good	0.869 (0.676–1.118)	0.774 (0.502–1.194)	1.156 (0.924–1.446)
Non-smartphone screen time (h/day)		1.045 (0.998–1.095)	0.948 (0.861–1.044)	0.982 (0.918–1.050)
PSU for social network (out of 30 points)^‡,d^		0.994 (0.967–1.021)	1.005 (0.960–1.053)	0.986 (0.963–1.008)
PSU for entertainment (out of 20 points)		1.030 (1.005–1.057)^*^	0.995 (0.947–1.045)	1.031 (1.004–1.059) ^*^
PSU for information collection (out of 15 points)		0.998 (0.970–1.027)	1.015 (0.951–1.083)	1.006 (0.966–1.046)

*P < 0.05; **P < 0.01; ***P < 0.001

† *PA* Physical activity. ‡ PSU: Problematic smartphone use

a Participants with non-zero smartphone screen time were recruited

b Measured on a five-point scale with one question: “Would you say that your study pressure is very much, much, moderate, a little, or none?”

c Measured on the Chinese version of the World Health Organization’s Five-item Well-being Index (WHO-5)

d Dimensions of problematic smartphone use (social network, entertainment, and information collection) were measured on the Revised Problematic Smartphone Use Classification Scale

**Table 5:** Odds ratios (OR) and 95% confidence intervals (95% CI) of predictors of overweight/obesity in participants by sex^a,b^

Variables		Model D: *n* = 2549 Male OR (95% CI)	Model E: *n* = 2748 Female OR (95% CI)
Educational stage	Primary school	Reference	Reference
	Middle school	0.605 (0.391–0.935)^*^	2.627 (1.539–4.486)^***^
	High school	1.033 (0.502–2.124)	5.482 (2.158–13.926)^***^
Age		0.942 (0.840–1.057)	0.751 (0.649–0.870)^***^
Indoor PA time (m/day)^†^	< 30	Reference	Reference
	30–60	1.033 (0.845–1.262)	1.014 (0.809–1.271)
	> 60	0.933 (0.735–1.184)	0.993 (0.727–1.357)
Outdoor PA time (m/day)	< 30	Reference	Reference
	30–60	0.971 (0.781–1.207)	0.983 (0.770–1.257)
	> 60	0.967 (0.765–1.223)	0.891 (0.674–1.179)
Academic stress^b^		1.026 (0.945–1.114)	1.013 (0.906–1.133)
Mental health^c^	Poor	Reference	Reference
	Good	0.933 (0.762–1.142)	1.082 (0.848–1.381)
Non-smartphone screen time (h/day)		1.007 (0.967–1.049)	1.041 (0.976–1.110)
PSU for social network (out of 30 points)^‡,d^		0.997 (0.977–1.017)	0.980 (0.953–1.006)
PSU for entertainment (out of 20 points)		1.020 (0.998–1.042)	1.046 (1.018–1.075)^**^
PSU for information collection (out of 15 points)		0.999 (0.972–1.026)	1.005 (0.970–1.042)

*P < 0.05; **P < 0.01; ***P < 0.001

† *PA* Physical activity. ‡ PSU: Problematic smartphone use

a Participants with non-zero smartphone screen time were recruited

b Measured on a five-point scale with one question: “Would you say that your study pressure is very much, much, moderate, a little, or none?”

c Measured on the Chinese version of the World Health Organization’s Five-item Well-being Index (WHO-5)

d Dimensions of problematic smartphone use (social network, entertainment, and information collection) were measured on the Revised Problematic Smartphone Use Classification Scale

## Discussion

This study revealed that the overweight and obesity rates in male students (20.7 and 16.4%) were higher than those in female students (11.2 and 8.2%), a finding that is consistent with those of other studies in China [41, 42], as well as among Japanese [43], South Korean [44], and Chinese-American children and adolescents [45].

Regarding problematic smartphone use, the scores were significantly different in all dimensions by the educational stage. Our study found that middle and high school students scored higher on the total problematic smartphone use than primary school students. This is consistent with other studies involving students of different grades in smartphone addiction among Chinese school students [46]. In terms of sex differences, problematic smartphone use in male students was significantly more common than that in female students; however, the overall and dimension-specific problematic smartphone use scores were still below the mid-score. Our study results here were consistent with a study in India and another study in China [47, 48], but different from a study in South Korea [49]; said study from South Korea instead found that there were more females in the smartphone addiction group, while certain other research studies reported no significant association between sex and problematic smartphone use [50, 51]. This discrepancy might be explained by the fact that our content-based scale had an emphasis on smartphone use in the context of Internet consumption and on considering specific usage purposes (social networks, entertainment, and information collection), which might be different from the components of general smartphone dependence scales used by other studies. Due to the various evaluation tools available for problematic smartphone use, it is hard to compare our results with previous research accurately.

According to the bivariate analysis, overall problematic smartphone use and problematic smartphone use in the dimension of entertainment showed a positive correlation with overweight or obesity. Consistent with our findings, several Internet and cellular phone-related activities were found to be associated with increased BMI in a study of adolescents in Taiwan [52]. To be specific, our multivariate logistic regression analysis revealed that only problematic smartphone use for entertainment was positively related to obesity status among students from primary and high school. Interestingly, no significant correlation between problematic smartphone use and obesity status was discovered among students from middle school; this might be explained by the fact that students’ self-control increased with age, so students from middle school were not as susceptible to problematic smartphone use as those from primary school. Moreover, our study showed that students’ academic stress was significantly lower in middle school than that in high school, which might decrease the risk for middle school students to display problematic smartphone use since previous studies had proven the existence of a positive association between stress and smartphone addiction [34]. For students from primary school and high school, problematic smartphone use for entertainment might reduce their physical activity (especially outdoor physical activity) and lengthen their sedentary time, thus causing an increase in obesity status.

The relationship between problematic smartphone use and obesity status also differed by sex. Our study showed that problematic smartphone use for entertainment was significantly associated with obesity status for female students, and the association was positive. Their problematic smartphone use for entertainment might be more for reading online novels or binge-watching videos, which might take more sedentary time than playing online games. This kind of overuse requires sedentary behavior and, therefore, might account for the obesity status increase.

However, the association between problematic smartphone use and obesity was weak though statistically significant, which was consistent with a study of 4098 adolescent Finnish twins in which the monthly smartphone bill was adopted to address the smartphone use [26]. One possible reason for the weak association was that the problematic smartphone use was treated as a continuous variable instead of a categorical variable in this study due to the absence of classification criteria of problematic smartphone use. The other was that there were mediator variables that might strengthen the association but were not collected as covariates, such as dietary habits, sleep quality, etc. Contrary to our findings, a study of 482 children with the average age of 12 years old showed that information technology use, especially Internet use, smartphone use, and videogame playing, did not predict BMI or bodyweight [53]. The contradictory results might be explained by the different forces of external supervision for smartphone use and different academic stress. Students with less academic stress tended to have sufficient leisure time, which might ensure physical activity time in spite of problematic smartphone use. Additionally, the association between problematic smartphone use and obesity might vary with the popularity of the smartphone, which was determined by the level of economic and social development. The demographic characteristics of the target population might also influence the result, especially age and occupation. A prospective randomized study of 440 patients with overweight or obesity in Iraq, whose target population was different from our study, revealed that excessive use of smartphones might be a potential factor in the initiation of overweight or obesity [25]. In addition, previous studies have shown that excessive smartphone use can impair physical health (e.g., lead to vision loss and musculoskeletal problems) [54], while prompting mental health problems (e.g., depression and anxiety) [55], maladjustments at school [56], compromised privacy and cyberbullying [57]. Therefore, we should:(1). To help reduce the excessive use of smartphones, physical activity and outdoor activities should be encouraged in children and adolescents by increasing physical education class hours and sports facilities on campus. (2). Encourage families to develop and adhere to a Family Media Use Plan, which addresses what type of media and how much is appropriate for each child, while promoting that they get the recommended amount of physical activity, sleep and study. (3). Encourage parents to talk to their children about the concept of being a ‘good digital citizen’ and to discuss the serious consequences of online or cyberbullying. (4). Promote continued research into risks and benefits of media and disseminate findings to families and educators [57].

There were limitations to our study. First, participants’ physical examination data were obtained from existing school records instead of onsite measurements. Despite this, the measures of physical examination had been validated. Second, the direction of the causal relationship could not be clarified due to the natural limitations of a cross-sectional study. Third, the measurements of physical activities, mental health, academic stress were based on self-reports instead of validated questionnaires, and the items in the survey (e.g., the estimation of physical activity time) might be difficult for primary and middle school students to interpret or assess. However, in our study, self-reports might be the most accurate way of assessing mental health and academic stress, given that individuals would have better insight into their own psychological characteristics than outside observers would [58]. Fourth, dietary habits represented an essential influencing factor for childhood obesity; however, information on such mediator variables was not collected. Although this study proves that obesity in children and adolescents is associated to smartphone use, future research also needs to determine the direction of the correlation between obesity status and problematic smartphone use. Because some children with obesity may be living with one of the many known co-morbidities of obesity, which affects their mobility or desire to participate in physical activities (such as musculoskeletal issues or pain), or indeed experience the related general stigma of overweight and obesity which prevents their participation in activities, and as a result, their smartphone usage increases. And using objective measurements of physical activities (e.g., wearable movement-tracking sensors) and problematic smartphone use (e.g., custom behavior-tracking app).

## Conclusion

In general, our study examined the association between problematic smartphone use and overweight/obesity in children and adolescents, revealing different associations in dimensions by educational stage and sex. With a large sample size, content-based problematic smartphone use evaluation scale, and adjustment of potential obesity status influencing covariates, the results of our study are convincing and thus provide a scientific reference for practical interventions to control obesity in a school-age population. Healthier ways of entertainment should be introduced to primary and high school students, and limiting smartphone usage should be considered as an integrated component of pediatric weight management interventions. In terms of sex differences, to control the prevalence of overweight and obesity, female students in particular may be susceptible to problematic smartphone use and preventative measures tailored for girls may help to address this issue.

## Supplementary Information

**Additional file 1: Table S1.** Distribution of the participants in different educational stages and districts.

**Additional file 2: S2.** The Development of Revised Problematic Smartphone Use Classification Scale (RPSUCS).

**Additional file 3: Table S3.** Revised Problematic Smartphone Use Classification Scale (RPSUCS).

## Data Availability

All data generated or analysed during this study are included in this published article.

## Abbreviations

BMI: body mass index
OR: odds ratio
CI: confidence interval
COVID-19: coronavirus disease 2019
WGOC: Working Group on Obesity in China
RPSUCS: Revised Problematic Smartphone Use Classification Scale
PSUCS: Problematic Smartphone Use Classification Scale
ICC: Interclass Correlation Coefficient
PA: Physical activity
PSU: Problematic smartphone use
