# Supplementary material for: The association between obesity and problematic smartphone use among school-age children and adolescents: a cross-sectional study in Shanghai
Source: BMC Public Health. 2021 Nov 11;21:2067. doi: 10.1186/s12889-021-12124-6 (PMC8581960; doi:10.1186/s12889-021-12124-6)
Supplement: Supplementary file 1 — Additional file 1: Table S1. Distribution of the participants in different educational stages and districts. [file 12889_2021_12124_MOESM1_ESM.pdf]

Table S1 Distribution of the participants in different educational stages and districts

Location	Number of participants			Total
	Primary school	Middle school	High school	
Urban_1	343	0	0	343
Urban_2	307	216	0	523
Urban_3	369	0	418	787
Urban_4	516	0	0	516
Urban_5	137	0	407	544
Urban_6	207	112	207	526
Semi-urban and semi-suburban _1	879	214	463	1556
Suburban_1	0	242	226	468
Suburban_2	217	0	199	416
Suburban_3	211	0	0	211
Suburban_4	220	0	184	404
Suburban_5	195	0	207	402
Suburban_6	217	210	223	650
Suburban_7	208	0	103	311
Suburban_8	703	0	0	703
Total	4729	994	2637	8360
