# Supplementary material for: The association between obesity and problematic smartphone use among school-age children and adolescents: a cross-sectional study in Shanghai
Source: BMC Public Health. 2021 Nov 11;21:2067. doi: 10.1186/s12889-021-12124-6 (PMC8581960; doi:10.1186/s12889-021-12124-6)
Supplement: Supplementary file 2 — Additional file 2: S2. The Development of Revised Problematic Smartphone Use Classification Scale (RPSUCS). [file 12889_2021_12124_MOESM2_ESM.pdf]

S2 The Development of Revised Problematic Smartphone Use Classification Scale (RPSUCS)

1 Problematic Smartphone Use Classification Scale (PSUCS)

Table 1 showed the items of PSUCS, which were adopted to measure problematic smartphone use (PSU) among Chinese middle school students. The RPSUCS for Chinese school-age children and adolescents was developed based on PSUCS.

Table 1 Problematic Smartphone Use Classification Scale (PSUCS)

Dimension	Item
PSU on social network	1. I spend a lot of time on a daily basis on social apps (QQ, WeChat, Weibo, etc.).
	2. I usually have a late bedtime after using social apps in bed.
	3. I spend more and more time on social apps.
	4. Because of chatting on the Internet, parents and friends have opinions about me, but I still haven't reduced the time for online chat.
PSU on entertainment	5. I enjoy using entertainment apps, e.g. playing mobile games, watching videos, reading novels, etc.
	6. Using entertainment apps is my favorite way to relax and reduce stress.
	7. I always keep up with updates from online novels and/or games.
	8. I spend a lot of time on mobile entertainment.
	9. I have a strong sense of achievement and satisfaction after completing a game, video series or novel.
PSU on compulsive behavior	10. I cannot resist the impulse to check for message updates in social apps (QQ, WeChat, Weibo, etc.).
	11. I always reply to the news on social apps, even sneaking back during class, otherwise I will feel uneasy
	12. Even thinking about using entertainment apps (gaming, video, novel, etc.) distracts me from other activities.
PSU on information collection	13. I will read the news pushed by the mobile phone apps in the first time.
	14. I spend a lot of time on a daily basis searching for and reading various news and information.
	15. If I don't read news, I will feel restless.

2 Development of RPSUCS

2.1 Validity

2.1.1 Exploratory Factor Analysis (EFA)

EFA was conducted in the results of 8 419 students by Principal Component Analysis and Varimax Rotation. Items that met any of the following conditions would be deleted: (1) the items of which factor loads on all dimensions being less than 0.6; (2) the items of which factor loads being high on two or more dimensions while the differences of the factor loads being less than 0.1; (3) the items of which the number in one dimension being less than 3.

In the first EFA, the Kaiser-Meyer-Olkin (0.920) and Bartlett's tests ($p<0.001$) showed that the data was suitable for factor analysis. A three-factor structure was extracted from the first EFA. The factor loads of item 8 on the three extracted dimensions were 0.593, 0.553, and 0.055, while those of item 12 were 0.598, 0.217, and 0.309. Therefore, item 8 was deleted because it met the first and second conditions mentioned above, while item 12 was deleted according to the first condition.

The second EFA was conducted after the item 8 and item 12 were deleted, and it extracted a three-factor structure accounting for 65.074% of the total variance. Factor 1, "PSU on social network," included six items (item 1, 2, 3, 4, 10, 11). Factor 2, "PSU on entertainment," included four items (item 5, 6, 7, 9). Factor 3, "PSU on

information collection," included three items (item 13, 14, 15) (Table 2).

Table 2 Principal Components Analysis results of RPSUCS

Item	Component		
	1	2	3
Item 2	**0.797**	0.236	0.037
Item 1	**0.755**	0.297	0.067
Item 3	**0.752**	0.320	0.061
Item 4	**0.725**	0.302	0.074
Item 10	**0.667**	0.140	0.242
Item 11	**0.663**	0.053	0.306
Item 6	0.201	**0.833**	0.102
Item 5	0.215	**0.823**	0.074
Item 9	0.247	**0.761**	0.160
Item 7	0.326	**0.713**	0.132
Item 14	0.107	0.121	**0.829**
Item 15	0.121	0.050	**0.809**
Item 13	0.164	0.170	**0.733**
% of variance	42.023	12.816	10.235
Cumulative % of variance	42.023	54.839	65.074

2.1.2 Confirmatory Factor Analysis (CFA)

The proposed three-factor model of the RPSUCS was tested by computing CFA with AMOS 22.0. According to the modification indices, residuals e5 and e6 were linked to amend the model (Figure 1).

The overall model fit was judged by the following cut-off values: For the Normed Fit Index (NFI), Relative Fit Index (RFI), Incremental Fit Index (IFI), Tucker-Lewis Index (TLI), and Comparative Fit Index (CFI), values larger than 0.95 indicated a good model fit while values between 0.90 and 0.95 indicated an acceptable model fit;

for the Root Mean Square Error of Approximation (RMSEA), values smaller than 0.05 indicated a good fit, values between 0.05 and 0.08 indicated an acceptable fit and values greater than 0.10 indicated a poor model fit. The following Goodness of Fit indices indicated a good fit of the model to the data: Chi-square ($\chi2$) = 1490.915, $p < 0.001$, NFI = 0.969, RFI = 0.960, IFI = 0.970, TLI = 0.962, CFI = 0.970, and RMSEA = 0.053.

Figure 1 Three-factor model of the RPSUCS

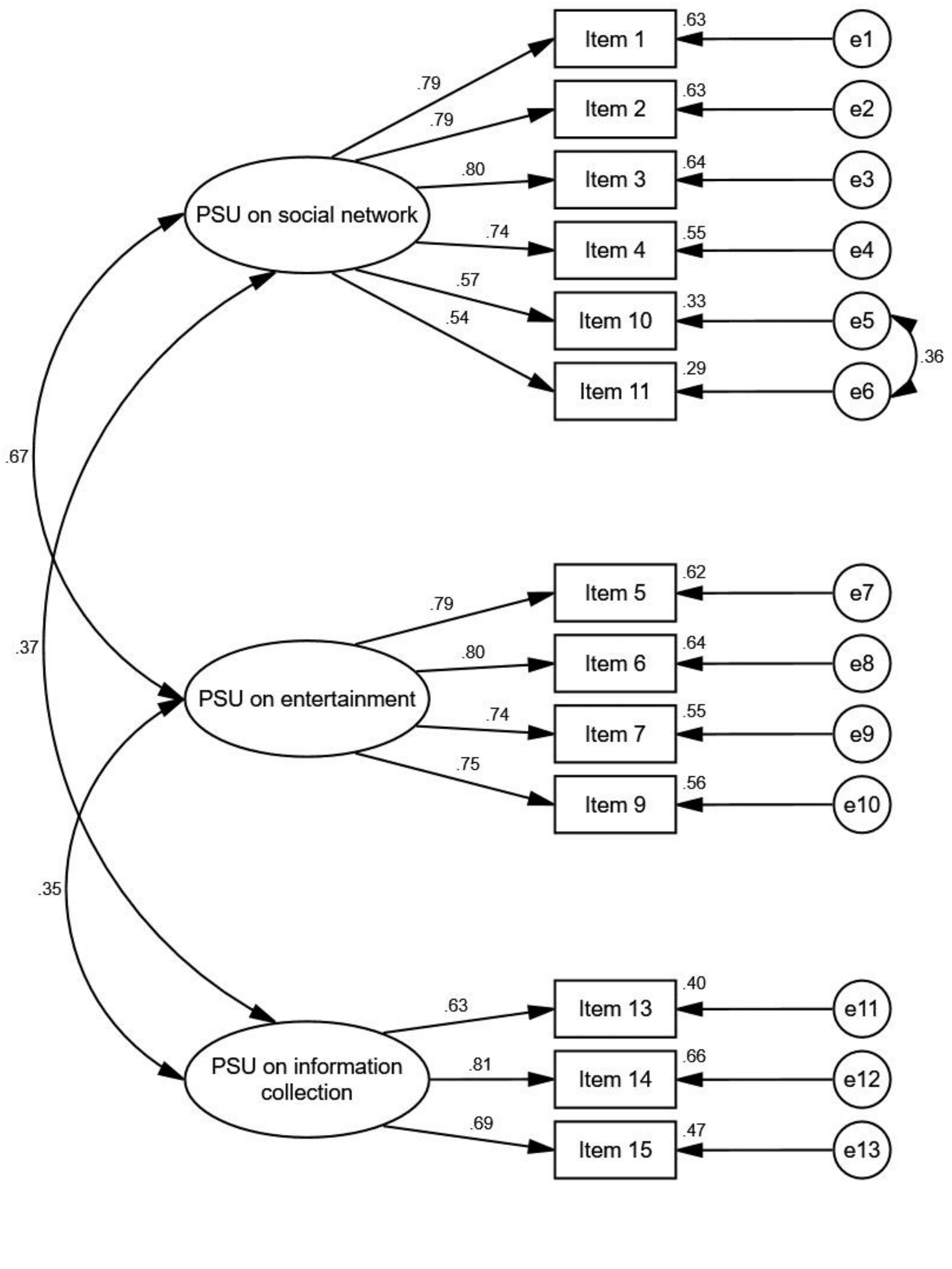

2.2 Reliability

2.2.1 Internal consistency reliability

Internal consistency reliability was measured using the Cronbach α coefficient. The Cronbach's α coefficient of the total scale, PSU on social network, PSU on

entertainment, and PSU on information collection respectively, were 0.876, 0.863, 0.852, and 0.750.

2.2.2 Test-retest reliability

A sample of 540 participants was retested to assess the test-retest reliability of RPSUCS two months later. Test-retest reliability was indicated by the interclass correlation coefficient (ICC) of total score and score of three dimensions at the two tests. The ICCs of total scale, PSU on social network, PSU on entertainment, and PSU on information collection were 0.763, 0.850, 0.645, and 0.609, respectively.

2.3 Final version of RPSUCS

The above analysis dementated that RPSUCS had satisfactory validity and reliability. The items of RPSUCS were rearranged and exhibited in Table 3.

Table 3 Revised Problematic Smartphone Use Classification Scale (RPSUCS)

Dimension	Item
PSU on social network	1. I spend a lot of time on a daily basis on social apps (QQ, WeChat, Weibo, etc.).
	2. I usually have a late bedtime after using social apps in bed.
	3. I spend more and more time on social apps.
	4. Because of chatting on the Internet, parents and friends have opinions about me, but I still haven't reduced the time for online chat.
	5. I cannot resist the impulse to check for message updates in social apps (QQ, WeChat, Weibo, etc.).
	6. I always reply to the news on social apps, even sneaking back during class, otherwise I will feel uneasy
PSU on entertainment	7. I enjoy using entertainment apps, e.g. playing mobile games, watching videos, reading novels, etc.
	8. Using entertainment apps is my favorite way to relax and

	reduce stress.
	9. I always keep up with updates from online novels and/or games.
	10. I have a strong sense of achievement and satisfaction after completing a game, video series or novel.
PSU on information collection	11. I will read the news pushed by the mobile phone apps in the first time.
	12. I spend a lot of time on a daily basis searching for and reading various news and information.
	13. If I don’t read news, I will feel restless.
