# Supplementary material for: The association between obesity and problematic smartphone use among school-age children and adolescents: a cross-sectional study in Shanghai
Source: BMC Public Health. 2021 Nov 11;21:2067. doi: 10.1186/s12889-021-12124-6 (PMC8581960; doi:10.1186/s12889-021-12124-6)
Supplement: Supplementary file 3 — Additional file 3: Table S3. Revised Problematic Smartphone Use Classification Scale (RPSUCS). [file 12889_2021_12124_MOESM3_ESM.pdf]

Table S3 Revised Problematic Smartphone Use Classification Scale (RPSUCS)

Dimension	Item
Problematic smartphone use (PSU) on social network	1. I spend a lot of time on a daily basis on social apps (QQ, WeChat, Weibo, etc.).<br>2. I usually have a late bedtime after using social apps in bed.<br>3. I spend more and more time on social apps.<br>4. Because of chatting on the Internet, parents and friends have opinions about me, but I still haven't reduced the time for online chat.<br>5. I cannot resist the impulse to check for message updates in social apps (QQ, WeChat, Weibo, etc.).<br>6. I always reply to the news on social apps, even sneaking back during class, otherwise I will feel uneasy
PSU on entertainment	7. I enjoy using entertainment apps, e.g. playing mobile games, watching videos, reading novels, etc.<br>8. Using entertainment apps is my favorite way to relax and reduce stress.<br>9. I always keep up with updates from online novels and/or games.<br>10. I have a strong sense of achievement and satisfaction after completing a game, video series or novel.
PSU on information collection	11. I will read the news pushed by the mobile phone apps in the first time.<br>12. I spend a lot of time on a daily basis searching for and reading various news and information.<br>13. If I don't read news, I will feel restless.
